# Presence of Eyelid Disease in Habitual Scleral Lens Wearers

**DOI:** 10.3390/jcm15093181

**Published:** 2026-04-22

**Authors:** Jennifer S. Harthan, Amy Nau, Ellen Shorter, Cherie B. Nau, Muriel Schornack, Jennifer Swingle Fogt

**Affiliations:** 1 Cornea and Contact Lens Center of Clinical Excellence, Illinois College of Optometry, Chicago, IL 60025, USA; jharthan@ico.edu; 2 Forefront Eye Care, Boston, MA 02111, USA; anau@forefronteyecare.com; 3 Department of Ophthalmology and Visual Sciences, University of Illinois at Chicago, Chicago, IL 60612, USA; eshorter@uic.edu; 4 Mayo Clinic Minnesota, Rochester, MN 55905, USA; schornack.muriel@mayo.edu; 5 College of Optometry, The Ohio State University, Columbus, OH 43210, USA; fogt.78@osu.edu

**Keywords:** scleral lenses, meibomian gland dysfunction, dry eye disease, eyelid disease

## Abstract

**Background/Objectives**: As scleral lens (SL) use continues to expand, particularly for medically necessary management of corneal irregularity and ocular surface disease, it is important to determine if wear of these lenses is associated with eyelid disease. This study evaluated habitual SL wearers for meibomian gland obstruction, meibum quality, and signs of lid margin inflammation. **Methods**: Eligible participants wore scleral lenses in one or both eyes for any indication and had been wearing their current lens design(s) for at least six months, with habitual wear of ≥5 h per day, six days per week. Eyelid inflammation metrics were specifically assessed. **Results**: Forty-nine scleral lens wearers (32 females) were enrolled. Upper lid wiper epitheliopathy (LWE) was present in 21 eyes (43%), and lower LWE was present in 15 eyes (31%). Thirty-nine eyes (80%) had expressible meibomian glands. Eyes with no expressible meibum were significantly higher in ocular surface disease (39%, 7/18) than corneal irregularity (11%, 3/27) (*p* = 0.03). Lid margin telangiectasia was present in wearers with ocular surface disease (61%, 11/18) and corneal irregularity (19%, 5/26) (*p* = 0.005). **Conclusions**: Eyelid disease was common among habitual scleral wearers regardless of indication for lens use. Eyes with ocular surface disease demonstrated a higher prevalence of meibomian gland obstruction and lid margin telangiectasia compared to those with corneal irregularity.

## 1. Introduction

A healthy pre-ocular tear film is necessary for the health of the ocular surface. This dynamic structure is the consequence of a highly coordinated effort between multiple tissue types which maintain homeostasis of the ocular surface, regulate inflammatory pathways, and provide clear vision. Structures contributing to the tear film include the cornea, the primary and accessory lacrimal glands, the conjunctiva and meibomian glands, the eyelids, and the neural communication structure connecting these sub-units. Dysfunction can affect any or several of these structures, resulting in various subtypes of ocular surface disease (OSD). The overwhelming majority of dry eye patients suffer from evaporative dry eye, followed by autoimmune conditions affecting the lacrimal gland. A much smaller percentage of patients suffer from conditions affecting the goblet cells. These subtypes can co-exist within the same patient.

Successful contact lens use depends on a healthy tear film and ocular surface. Because contact lenses disrupt tear film architecture and interact with the conjunctiva and eyelids, they can contribute to inflammation [1]. Scleral lenses (SLs) are widely prescribed for irregular corneas and severe ocular surface disease, yet their impact on eyelid structures essential for maintaining tear film integrity remains unclear [2,3,4,5]. Meibomian gland dysfunction (MGD) is a chronic and highly prevalent condition and is the leading cause of ocular surface disease (OSD) and contact lens discomfort [2,6,7,8]. The meibomian glands, located along the posterior eyelid, secrete the lipid layer of the tear film, which is critical for reducing tear evaporation. The 2011 International Workshop on MGD defined the condition as a chronic abnormality of the meibomian glands, often involving duct obstruction and altered meibum quality or quantity [7]. Previous studies have shown that both soft and rigid contact lenses can exacerbate meibomian gland dropout, alter gland morphology, reduce meibum quality and expressibility, and increase dry eye symptoms [9,10,11]. Because scleral lenses also interact with the eyelids and tear film, they may similarly have adverse effects on meibomian gland health. Several clinical features of OSD can be present in patients who wear SLs. These include MGD, lid wiper epitheliopathy, and lid margin telangiectasia. The extent to which these conditions are present in patients who wear scleral lenses is not known.

The presence of telangiectatic vessels along the lid margin is abnormal and serves as a clinical biomarker for chronic inflammation. Multiple molecular mechanisms contribute to these vascular changes, including degradation of vessel architecture, increased permeability, angiogenesis, and enhanced migration of inflammatory cells into the surrounding tissue [12]. Telangiectasia has been associated with MGD, blepharitis, bacterial biofilms, toxicity from preserved topical medications, cosmetic use, and Demodex infestation.

Humans blink more than 4000 times per day, and the “lid wiper” region of the eyelids is in constant contact with the ocular surface. Increased friction between the eyelid and the ocular surface or a contact lens can lead to alterations of the marginal palpebral conjunctiva [13]. These changes include focal microvascular alterations, histological enlargement of the lid wiper region, and increased keratinization [13,14]. Lid wiper epitheliopathy is a well-established complication of contact lens wear [15] and is also a sign of ocular surface disease [16].

The two primary indications for scleral lens wear are corneal irregularity and ocular surface disease [17]. As scleral lens use continues to expand, particularly for managing ocular surface disease, it is important to determine if wear of these lenses is associated with eyelid disease. This study evaluated habitual scleral lens wearers for meibomian gland obstruction, meibum quality, and signs of lid margin inflammation.

## 2. Materials and Methods

This multi-center, prospective observational study was approved by The Ohio State University Institutional Review Board and by each participating site. Data were collected at five clinical locations: the Ohio State University, Columbus, OH; Illinois College of Optometry, Chicago, IL; University of Illinois at Chicago, Chicago, IL; Mayo Clinic, Rochester, MN; ForeFront Eye Care, Boston, MA. Written informed consent was obtained from all participants, and all procedures adhered to the Declaration of Helsinki. Investigators at all sites collaborated on the study design and were aligned on all examination and data collection completed during the study.

Established scleral lens wearers were recruited for this study. Eligible participants >18 years of age wore scleral lenses in one or both eyes for any indication and had been wearing their current lens design(s) for at least six months, with habitual wear of ≥5 h per day, at least six days per week. On the study visit day, participants were required to have worn their lenses for at least two hours before being examined. Seven investigators performed clinical assessments: two each at Mayo Clinic and the Ohio State University, and one at each remaining site. Demographic information was collected, including age, sex, ethnicity, and indication for SL wear. Lens wear time, duration of scleral lens wear experience, lens diameter, and haptic design were collected for each participant.

A slit lamp evaluation assessed ocular health and lens fit according to established protocols. The fit of the scleral lens was assessed, specifically examining for adequate vault over the cornea and limbus. The alignment of the landing zones of the lenses was assessed and any areas of conjunctival capillary impingement or compression under the lens were noted. After removal of the scleral lens, ocular structures were examined for specific contact lens-related complications including bulbar conjunctival rebound vasodilation, bulbar, tarsal and corneal fluorescein staining, and corneal epithelial swelling, which are all standard for fit assessment. Eyelid inflammation metrics were specifically assessed. Meibomian gland secretion was evaluated by applying gentle manual pressure vertical to the base of the eyelashes with the index finger at the central lower lid margin. Presence or absence of meibomian gland secretion was documented; if present, secretion quality was graded as clear, turbid, or solid. Next, the presence or absence of lid wiper epitheliopathy was noted following instillation of fluorescein. If widening of the mucocutaneous junction of the upper and/or lower lid was detected by vital dye staining, lid wiper was graded as present. Finally, the absence or presence of lid margin telangiectasia on the upper and lower lid margin was recorded. Study data were collected and managed using REDCap electronic data capture tools hosted at the Ohio State University [18,19]. No masking of indication for lens wear occurred in this study.

Descriptive and comparative statistics were conducted using Minitab (Version 21.3.1; Minitab, LLC, State College, PA, USA). For participants wearing scleral lenses bilaterally, one eye was randomly selected for analysis using a random number generator. Pearson chi-square tests were used to compare lid margin telangiectasia, lid wiper epitheliopathy, absence of expressible meibum, and meibum quality in eyes by indication for scleral lens wear (comparing eyes with corneal irregularity vs. eyes with ocular surface disease) and by presence or absence of reported midday fogging. A two-sample *t* test was used to compare mean participant age, daily lens wear time, duration of scleral lens wear, and lens diameter worn in the corneal irregularity and ocular surface disease eyes.

## 3. Results

Forty-nine scleral lens wearers (32 females) were enrolled. The mean age was 49 ± 14 years (range 21–77). Indications for scleral lens wear included corneal irregularity (55%, 27/49), ocular surface disease (39%, 18/49), and four participants (8%) classified as “other” (three wore scleral lenses for refractive correction; one for neurotrophic keratitis). One eye per participant was analyzed.

Overall, upper lid wiper epitheliopathy was present in 21 eyes (43%), and lower lid wiper epitheliopathy was present in 15 eyes (31%). Thirty-nine eyes (80%) had expressible meibomian gland secretion: 29 clear, nine turbid, and one solid.

Clinical comparisons were made between the participants with corneal irregularity (CI) and ocular surface disease (OSD). The corneal irregularity group included 20 eyes (74%, 20/27) with keratoconus, and seven eyes with corneal irregularity not attributed to keratoconus. The ocular surface disease group consisted of participants fit with scleral lenses specifically for the purpose of treating dry eye and included three eyes (17%, 3/18) with ocular graft-vs.-host disease and two eyes (11%, 2/18) with Sjogren’s disease.

The mean ± standard deviation participant age was 47 ± 15 years for the CI group and 54 ± 15 years for the OSD group, which was not statistically significant (*p* = 0.2). The CI group consisted of 17 females (63%, 17/27) and the OSD group consisted of 11 females (61%, 11/18). The mean ± standard deviation daily wear time of the CI group (12.2 ± 3.0 h) and the OSD group (12.8 ± 2.6 h) were not statistically significant (*p* = 0.4). The duration of lens wear was also not statistically different when comparing the CI group (12 ± 8 years) to the OSD group (17 ± 11 years) (*p* = 0.1).

The designs of the habitual lenses worn by participants included five spherical (19%), seven toric (26%), and 15 (56%) quadrant-specific landing zones among the CI group and three (17%) spherical, two (11%) toric, and 13 (72%) quadrant-specific landing zone designs in the OSD group. The mean ± standard deviation of the lens diameters used in the CI group (17.4 ± 1.1 mm) and the OSD group (17.9 ± 1.1 mm) used were not statistically significant (*p* = 0.2).

Clinical findings consistent with MGD were observed in scleral lens wearers with both CI and OSD. Comparisons between CI (n = 27) and OSD (n = 18) groups found that the proportion of eyes with no expressible meibum was significantly higher in OSD (39%, 7/18) than CI (11%, 3/27) (*p* = 0.03). Lid margin telangiectasia was present in wearers with OSD (61%, 11/18) and CI (19%, 5/26) (*p* = 0.005). Palpebral conjunctival staining occurred in 19% (5/27) of CI eyes and 0% (0/18) of OSD eyes (*p* = 0.02). No statistically significant differences were found when comparing the presence of upper lid wiper epitheliopathy in the CI (44%, 12/27) and OSD groups (44%, 8/18) nor when comparing the presence of lower lid wiper epitheliopathy in the CI (30%, 8/27) and OSD groups (33%, 6/18).

Midday fogging was reported in 59% of eyes overall, occurring in 48% (13/27) of CI eyes, 72% (13/18) of OSD eyes, and 75% (3/4) of eyes in the “other” category. The presence of lid margin telangiectasia was higher in eyes with reported fogging (44%, 12/27) than those without fogging (19%, 4/21), approaching significance (*p* = 0.059). No other clinical findings differed significantly between eyes with and without fogging. Detailed results of signs distributed by indication are shown in Table 1.

## 4. Discussion

Eyelid disease, including MGD, is a major contributor to dry eye symptoms and contact lens intolerance [20,21]. Normal gland function is essential for ocular surface health and visual comfort, and it is largely supported by the mechanical action of blinking [22]. In this study of habitual scleral lens wearers, 80% demonstrated expressible meibum with forceful expression. It is important to recognize that force generated with a blink is substantially lower than that generated by digital pressure; therefore, the presence of expressible meibum in this study reflects gland patency under physical force rather than physiologic secretion during blinking. The OSD group showed a significantly higher proportion of eyes with no expressible meibum (39% versus 11%), suggesting that gland obstruction contributes meaningfully to the OSD seen in these patients. The fact that 11% of the CI group had no expressible meibum is a reminder that scleral lens wearers may have more than one disease process at play, although the findings of this study cannot differentiate whether this finding existed before scleral lens fitting or developed during the duration of scleral lens wear. Regardless, these wearers would benefit from treatment for MGD.

Interestingly, the presence of lid wiper epitheliopathy was the same rate in the CI and OSD groups (44%) and nearly the same rate for the lower lids (30% CI vs. 33% OSD). Lid wiper epitheliopathy develops due to increased friction between the palpebral conjunctiva and the ocular surface or lens surface [23]. The mechanism causing the friction may occur for different reasons for scleral lens wearers depending upon their indication for lens wear. Although patients with OSD often have reduced tear volume, which might be expected to increase friction, individuals with corneal irregularity tend to wear scleral lenses with steeper sagittal depths. These designs successfully vault over and protect irregular corneas but may increase mechanical interaction with the upper eyelid. This friction could also be the reason that eyes with CI in this study had a higher presence of staining of the palpebral conjunctiva than found in eyes in the OSD group. Because lid wiper epitheliopathy is often a clinical sign of ocular surface disease in non-lens wearers [16], it is important for practitioners to rule out OSD among SL wearers exhibiting LWE so that practitioners can differentiate whether OSD management or lens fit/parameter changes would be the better management plan for these lens wearers.

Lid margin telangiectasia, a biomarker of chronic inflammation, was unsurprisingly common in a majority of the OSD participants (61%) in this study. It is notable that it was also found in 19% of those with corneal irregularity. Although telangiectasia can develop for multiple reasons, its presence also indicates a reason to evaluate for OSD, even in those patients not originally fit with scleral lenses for that indication. Because OSD contributes to contact lens discomfort and discontinuation, comprehensive management of MGD and lid inflammation is especially critical in patients who rely on habitual therapeutic lens wear.

In this study, signs generally associated with OSD, including the presence of lid margin telangiectasia and lid wiper epitheliopathy and the absence of expressible meibum, were found among participants who were originally fit with scleral lenses for CI. These findings are in agreement with prior research suggesting an association between contact lens wear and MGD [24,25]. As early as the 1980s, obstructive disease from desquamated epithelial cells was identified as a major factor in dryness and discomfort among lens-intolerant patients [24]. Subsequent studies have demonstrated that the number of functional meibomian glands declines with long-term contact lens wear, although causality has not been shown to date [25]. Whether lens wear contributes to MGD, MGD contributes to lens intolerance, or both conditions exacerbate each other remains unclear.

The existing literature addressing MGD specifically in scleral lens wearers is limited. A 2022 study by Garcia-Marques et al. reported no acceleration of gland dropout in SL wearers, although it lacked a non-SL control group [2]. Conversely, Bolac et al. found significantly greater gland loss in SL wearers compared with both keratoconus and healthy controls [26]. Interpretation of these findings is complicated by the fact that patients with keratoconus, for example, have been shown to exhibit more severe MGD than age- and sex-matched controls [27,28].

Research on the interaction between MGD and SL-wearing experience is emerging. Schornack et al. found that patients reporting midday fogging were more likely to also report redness, suggestive of inflammation, during SL wear [29]. Elevated concentration of inflammatory mediators has also been identified in the fluid reservoir of SLs worn by patients who experience fogging [30,31]. In addition to inflammatory markers, higher concentrations of non-polar lipids have been found in reservoirs of fogging-associated lenses [32]. These lipids may decrease reservoir clarity and deposit on the front surface of SLs, contributing to non-wetting and subsequently to patient-reported fogging. Together, these findings support the hypothesis that ocular surface inflammation and altered lipid expression may negatively affect the SL-wearing experience.

Because this study aimed to evaluate the presence of eyelid disease among habitual scleral lens wearers, information was not collected on the long-term duration of scleral lens wear among the participants, and therefore no conclusions can be made about whether the presence of eyelid inflammation progressed with lens wear or was related to the original indication for lens wear. The cross-sectional design of this study and absence of pre-fitting baseline measurements limits the ability to determine whether observed lid findings developed prior to or because of scleral lens wear. The findings of this study, coupled with other previous cross-sectional research that has associated contact lens wear and scleral lens wear duration with changes in meibomian glands, show a need for studies of eyelid morphology over a long duration of scleral lens wear [25,26]. To determine whether SLs directly influence eyelid structure and function, longitudinal studies beginning with lens-naïve individuals are needed. Future studies should incorporate objective structural and functional assessments, including serial meibography and standardized gland expression using a meibomian gland evaluator to mimic physiologic blink. The study findings also highlight the potential value of incorporating more comprehensive eyelid and ocular surface assessments into scleral lens fitting protocols.

## 5. Conclusions

Eyelid disease was common among habitual SL wearers regardless of indication for lens use. Eyes with ocular surface disease demonstrated a higher prevalence of meibomian gland obstruction and lid margin telangiectasia compared to those with corneal irregularity. However, as an observational, cross-sectional study of habitual SL wearers, it was not possible to determine whether the eyelid disease was present before patients began wearing scleral lenses. Future prospective studies would be beneficial to evaluate the coexistence of conditions. The findings in the present study underscore the importance of routinely evaluating the eyelids in SL wearers and proactively addressing underlying dysfunction prior to or during SL fitting to optimize comfort, visual performance, and long-term wear success.

## Figures and Tables

**Table 1:** Clinically observed signs compared to indication for lens wear.

Sign	All Eyes	Corneal Irregularity (CI)	Ocular Surface Disease (OSD)	Other	*p*-Value of CI vs. OSD ^
n	49	27	18	4	
Midday fogging (self-reported)	29/49 (59%)	13/27 (48%)	13/18 (72%)	3/4 (75%)	0.6
Lower lid wiper epitheliopathy	15/49 (31%)	8/27 (30%)	6/18 (33%)	1/4 (25%)	0.8
Upper lid wiper epitheliopathy	21/49 (43%)	12/27 (44%)	8/18 (44%)	1/4 (25%)	1
No expressible meibomian glans	10/49 (20%)	3/27 (11%)	7/18 (39%)	0/4 (0%)	0.03
Lid margin telangiectasia	16/48 (33%)	5/26 (19%)	11/18 (61%)	0/4 (0%)	0.005
Corneal staining	11/49 (22%)	7/27 (26%)	4/18 (22%)	0/4 (0%)	0.8
Bulbar conjunctival staining	7/49 (14%)	4/27 (15%)	3/18 (17%)	0/4 (0%)	0.9
Palpebral conjunctival staining	5/49 (10%)	5/27 (19%)	0/18 (0%)	0/4 (0%)	0.02

^ Pearson chi-square analysis.

## Data Availability

The raw data supporting the conclusions of this article will be made available by the authors on request.

## Abbreviations

The following abbreviations are used in this manuscript:

SL	Scleral lens
LWE	Lid wiper epitheliopathy
MGD	Meibomian gland dysfunction
OSD	Ocular surface disease
LMT	Lid margin telangiectasia
MGS	Meibomian gland secretion
CI	Corneal irregularity
